# Intensive Group Learning and On-Site Services to Improve Sexual and Reproductive Health Among Young Adults in Liberia: A Randomized Evaluation of *HealthyActions*

**DOI:** 10.9745/GHSP-D-16-00074

**Published:** 2016-09-28

**Authors:** Rebecca Firestone, Reid Moorsmith, Simon James, Marilyn Urey, Rena Greifinger, Danielle Lloyd, Lisa Hartenberger-Toby, Jewel Gausman, Musa Sanoe

**Affiliations:** a Population Services International, Washington, DC, USA; b Population Services International, Yangon, Myanmar; c Education Development Center, Inc., Washington, DC, USA; d Population Services International, Monrovia, Liberia; e Education Development Center, Inc., Pasig City, Philippines; f Independent Consultant, Harvard T.H. Chan School of Public Health, Boston, MA; g Education Development Center, Inc., Advancing Youth Project, Monrovia, Liberia

## Abstract

**Introduction:**

Young Liberians, particularly undereducated young adults, face substantial sexual and reproductive health (SRH) challenges, with low uptake of contraceptive methods, high rates of unintended pregnancy, and low levels of knowledge about HIV status. The purpose of this study was to assess the impact of a 6-day intensive group learning intervention combined with on-site SRH services (called *HealthyActions*) among out-of-school young adults, implemented through an existing alternative education program, on uptake of contraception and HIV testing and counseling (HTC).

**Methods:**

The intervention was implemented among young women and men ages 15–35 who were enrolled in alternative basic education learning sites in 5 counties of Liberia. We conducted a randomized evaluation to assess program impact. Baseline data were collected in January–March 2014, and endline data in June–July 2014. Key outcomes of condom use, contraceptive use, and HTC were estimated with difference-in-difference models using fixed effects. All analyses were conducted in Stata 13.

**Results:**

We assessed outcomes for 1,157 learners at baseline and 1,052 learners at endline, across 29 treatment and 26 control sites. After adjusting for potential confounders, learners in the *HealthyActions* intervention group were 12% less likely to report never using a condom with a regular partner over the last month compared with the control group (*P* = .02). Female learners who received *HealthyActions* were 13% more likely to use any form of modern contraception compared with learners in control sites (*P*<.001), with the greatest increase in the use of contraceptive implants. Learners in *HealthyActions* sites were 45% more likely to have received HTC (*P*<.001).

**Conclusion:**

Providing intensive group learning in a supportive environment coupled with on-site health services improved SRH outcomes among participating learners. The focus of *HealthyActions* on participatory learning for low-literacy populations presents an adaptable solution for health programming across Liberia and the region.

## Abstract (precis)

Combining intensive group learning and provision of on-site reproductive health services through an existing alternative basic education program increased use of contraception and HIV testing and counseling among young out-of-school Liberians.

## INTRODUCTION

Even before the recent Ebola crisis, Liberia has faced intersecting health and development challenges. Fourteen years of conflict disrupted preexisting social structures, resulting in young people being forced to take on adult responsibilities without experiencing healthy developmental milestones.^1^ Liberians value education highly as the path to better employment, and particularly appreciate the positive impact that educating girls can have on families and communities.^2^ However, due to war, poverty, and family disruption, many young people in Liberia were forced to drop out of school and never participated in community leadership structures. In a sense, they were caught between youth and adulthood.

As a result, in Liberia the government’s definition of youth—15–35 years—is unique to a post-conflict environment. This broad definition speaks to delayed social and cognitive development across generations, warranting creative solutions to reach young people and provide access to much-needed services.^3^

Liberia’s legacy of conflict, orphanhood, lack of social cohesion, and school drop-out, combined with early sexual debut, place young Liberians at increased risk of negative sexual and reproductive health (SRH) outcomes. According to Liberia’s 2013 Demographic and Health Survey, the median age at first sex among women 25–49 is 16.2 years. Nearly one-quarter (24%) of women ages 25–49 had sexual intercourse by age 15; about three-quarters (78%) by age 18; and over 90% by age 20. Among men ages 25–49, the median age at first sex is slightly older at 18.3 years. By age 20, 75% of men have had sex.^4^ By age 24, nearly 80% of women have begun childbearing.^4^ Only 25% of sexually active girls and young women (ages 15–29) use a modern contraceptive method, and about 30% have an unmet need for contraception.^4^ Overall, HIV prevalence is low in Liberia at 1.9% for adults 15–49. Among women, peak prevalence (3.6%) occurs in the 25–29 age group; among men, prevalence is highest (3.6%) among those 40–44. While nearly all Liberians have heard of HIV/AIDS and the majority know where to get tested, only 37% of 15–24-year-old women and 11% of 15–24-year-old men have ever been tested and have received their results.^4^

Liberia’s legacy of conflict combined with early sexual debut places young Liberians at increased risk of negative sexual and reproductive health outcomes.

Low education levels exacerbate this situation; in 2013, 33% of women 15–49 and 13% of men 15–49 reported they had never been to school.^4^ Primary school gross enrollment is only 52%, and nearly a third of all students enrolled in school drop out in the first grade.^5^ Low rates of school enrollment and poor quality of teaching result in few Liberians gaining actionable knowledge and skills.^6^

Youth in Liberia face several barriers that limit their access to formal health services. To begin, the armed conflict led to an overall collapse of the health care system characterized by the flight of health workers, looting, and destruction of health facilities and the desecration of roads.^7^ Even where health services do exist, young people often face additional barriers. As in many countries in the world, young people in Liberia face financial barriers in accessing services, unfriendly providers (particularly if they are seeking SRH services and are unmarried), and lack of privacy and confidentiality.^8^ Unequal gender norms mean that young women are less likely than young men to receive an education and make autonomous decisions.^9^ Social acceptance of intimate partner violence reflects a culture of male dominance.^10^ Intergenerational and transactional sex is widespread across wealth quintiles. Many young women engage in transactional relationships to secure school fees, clothing, and other commodities.^4^^,^^11^^,^^12^

Young people in Liberia transition into adulthood in an environment in which there is pervasive distrust of all institutions connected with the government, which includes the health system. This may be in large part due to the fact that quality of life has increased minimally since the end of the conflict in 2003, despite donor money and support from the United Nations.^13^^,^^14^ Many communities lack social cohesion, and in some cases, young people are actively marginalized from existing organizational structures within their communities.^15^ The lack of formal training opportunities and/or education,^1^ along with limited supportive family structure, prevent many young people from becoming actively involved in their communities. The inability to rationalize appropriately within a group and/or to socialize normally during their formative years creates enormous barriers for young people to conform to conventional social norms.^16^ Further, there are widely respected taboos around discussing sex and sexuality, particularly for young women. Yet young women face greater pressure to have sex earlier, due to their desirableness to older men and the fact that the exchange system of young women’s sexuality for material goods has become normalized.^12^ These normalizations, however, have not relieved the heath system actors of their judgment about young people, and young women in particular.^17^

Supportive family, school, and peer environments are often associated with improved health outcomes among young people.^18^ Connectedness to schools, community, and family can have a protective effect on SRH outcomes, such as age of sexual debut, number of sexual partners, and contraceptive use.^19^ Programs that aim to strengthen social ties through participation in basic education can promote positive engagement in learning, socialization, and recreation while also improving access to SRH information and promoting healthy behavior.^20^

Uptake of SRH services is strongest when interventions designed to bolster demand for services are linked directly to supply, and where there is community and social support^21^; for example, an evaluation of a program implemented in Ghana and Nigeria that provided both contraceptives within a school setting and referrals for other SRH services as part of a comprehensive SRH program targeting youth, showed improved contraceptive uptake among participants.^22^ Educational settings, whether formal or informal, can provide ample opportunities for improving access to SRH information and services for young people. Information and services are brought to learners, rather than learners needing to seek them out. Additionally, learners can access services at the same time as one another, reducing the stigma associated with seeking contraceptive and HIV services.^23^ In Liberia, reaching young adults with reproductive health information and services through an existing platform could help overcome barriers to accessing services and ensure that they receive high-quality, youth-friendly services while also establishing relationships with local health service providers.

Formal and informal educational settings can provide opportunities for improving access to sexual and reproductive health information and services for young people.

Evidence of effective SRH strategies remains limited in West Africa, despite the efforts of the Ouagadougou Partnership to expand access to family planning.^24^ We aimed to assess whether an intervention package consisting of intensive group learning and provision of on-site SRH services could increase use of modern contraception and HIV testing and counseling (HTC) services among out-of-school young adults in Liberia. We hypothesized that participation in an alternative basic education project where this program, called *HealthyActions*, was implemented would be associated with greater use of modern contraception among women, greater condom use among young women and men, and greater use of HTC services by women and men, compared with participation in an alternative basic education project where *HealthyActions* was not implemented.

## METHODS

### Intervention

*HealthyActions* was structured as a 6-day intensive “burst” of participatory and action-oriented SRH education activities packaged with on-site health services, designed to be easily integrated into an alternative basic educational setting; in this case, the U.S. Agency for International Development (USAID) Advancing Youth Project,^25^ an alternative basic education project implemented by the Education Development Center (EDC). The USAID Advancing Youth Project, which began in October 2011 and will end in June 2017, provides basic literacy and numeracy skills, social and leadership development, and livelihoods training for out-of-school Liberian youth ages 15–35 who have no or marginal literacy skills. The first round of *HealthyActions* implementation took place from August 2012 to July 2013 in Nimba and Montserrado counties as a pilot to gain feedback on the curriculum and approach. The second round of larger-scale implementation took place from November 2013 to May 2014 in Bong, Grand Bassa, Lofa, Montserrado, and Nimba counties.

Over three-quarters of Advancing Youth learners are women, most of whom have never been to school before. The median age is 29. Most learners report that they are living with a partner or spouse and that they have an average of 3 children. Only about 5% are adolescents under the age of 18; these adolescents take the same classes as older learners and are integrated into all activities including youth clubs and skills training.^26^ The main difference between older and younger participants in the program is their experience of war; however, both groups have issues with identity formation and positive role models, and poverty puts learners at risk for transactional sex and other risky behaviors.

The USAID Advancing Youth Project works through the Liberian Ministry of Education, using government schools and teachers, with the classes scheduled in the evening to make learning accessible to young people who have left the formal school system. In the Advancing Youth Project’s approach to alternative basic education, Level 1 is for students who have never been to school at all or have dropped out in grade 1 or 2. Level 2 is roughly equivalent to grades 3 and 4, and level 3 to grades 5 and 6. Students are placed in the appropriate level based on the results of a short literacy and math test at intake.

*HealthyActions* was implemented through a partnership between Population Services International (PSI) Liberia and EDC, and was originally funded in partnership between the 2 organizations; 50% from EDC via the Advancing Youth Project and 50% from a PSI innovation fund. The Advancing Youth Project’s life-skills module has been one of the most popular among learners because of its relevance and applicability; however, teachers are often uncomfortable addressing SRH topics with learners.^27^ *HealthyActions* provided the opportunity to bring in expert trainers to reinforce learning and to link learners directly to health services. Since the majority of learners are young women who never attended or dropped out of school due to pregnancy, topics related to contraception and parent and partner communication are of extreme interest.

*HealthyActions* was conceived as a package of educational and empowering SRH activities that would motivate learners to use health services, which were brought to them to try on-site. The program consisted of 15 hours of classroom-based modules delivered over the course of 5 sequential days, focused on building knowledge and improving attitudes and skills related to SRH. The package then culminated in a community-based celebration on the 6th and final day of the program, during which *HealthyActions* participants and their families and communities were invited for a day of music, food, and health services. The celebrations were held on-site, with government health workers providing contraceptive counseling and services, HTC, and referrals to local clinics for *HealthyActions* participants and other community members. At sites where classrooms were available, health care providers used the classrooms as private and confidential spaces to provide services. At sites where private indoor space was limited, tables and curtains were used to create private and clean areas for service delivery. Participants who required clinical follow-up (e.g., for a positive HIV test, insertion of an intrauterine device [IUD], continued injections) were provided with referrals to the nearest government health facility.

*HealthyActions* consisted of educational activities to motivate learners to use health services, which were brought to them to try on-site.

The *HealthyActions* curriculum was designed through an iterative and youth-centered process that included (1) insight gathering with learners; (2) curriculum development; (3) pilot testing with learners; and (4) adaptation and implementation. Additionally, PSI looked to USAID’s recently released Youth in Development Policy to ensure the program design aligned with the U.S. Government’s key priorities for youth initiatives.^28^ In line with the policy, *HealthyActions*:

- Used an assets-based approach that focused on building internal assets (e.g., self-esteem, communication and assertiveness skills, gender transformative attitudes) as well as external assets (e.g., access to education and social cohesion)
- Involved communities by engaging learners, their parents, and their community members in health service delivery and promotion on community celebration days
- Created opportunities for youth participation particularly through insight gathering, piloting the curriculum, and peer education opportunities
- Worked cross-sectorally by integrating SRH education and services within formalized (albeit nontraditional) education programming

To begin, PSI conducted an analysis of published and gray literature with a particular eye toward any discourse on Liberian youth’s assets and behavioral drivers. Results were used to support the design of a focus group discussion guide that was fielded through 2 group discussions with USAID Advancing Youth Project learners to glean insights about their knowledge, attitudes, and perceptions of SRH. Key insights from those discussions—such as misconceptions about the safety and efficacy of certain contraceptive methods, harmful gender norms, and negative attitudes toward condom use—were used to design curriculum content. Other insights related to young people’s hopes, aspirations, and curiosity were used to design curriculum delivery approaches.

The curriculum was grounded in the information, motivation, behavioral skills, and resources (IMBR) model, a health behavior theory often used in peer-based learning contexts that places equal emphasis on teaching young people knowledge and skills while also increasing access to products and services.^29^ Adult learning approaches were incorporated that included games and trivia to build knowledge; values exercises to address attitudes and norms; and role-play and small group discussions to drive critical thinking, self-esteem, and skill-building. Many of the curriculum’s activities drew on existing resources developed by international organizations, adapted for the Liberian context.

On Day 1, learners were introduced to the program and to one another, and they set group norms, took a pretest, and learned about “Healthy Competition”—a competitive element to the program. On that first day, the class was split into 2 teams. Every day thereafter, there was at least one competitive exercise whereby teams competed for points. At the end of the week, the team with the most points won a small prize, presented at the community celebration day. The competition provided a fun and engaging way to keep learners motivated and to improve teamwork.

Day 2 focused on puberty, sex, and reproductive health. Activities included body mapping exercises, true/false trivia games, role-playing exercises to practice talking about sex (with parents, health care workers, sexual partners, and their own children), and values exploration exercises to explore feelings about sexuality.

Day 3 focused on HIV/AIDS and included a game of tag to explain how HIV works in the body, true/false trivia, small group discussions about HIV stigma, and creating songs and dances to promote HTC.

Day 4 focused on contraception and pregnancy. Activities included a trivia game to learn about the available contraceptive methods, condom demonstration and practice, storyboard discussions about early pregnancy and community norms surrounding pregnancy outside of marriage, and values exploration surrounding use of contraception.

Day 5 focused on communication skills and taking action. Activities included a storyboard discussion on gender and power, small group discussions about coerced vs. voluntary sex, defining and practicing assertiveness skills, and a game to understand the negative effects of alcohol and drugs on decision making.^30^ Each day ended with a call to action (such as getting an HIV test or talking to a health care provider about contraception) and a condom demonstration, including practice putting condoms on models.

Day 6 was a clinic celebration day to mark the end of the curriculum and to introduce on-site health services to *HealthyActions* participants and members of the community. The aim in linking health service provision in a “burst” with the curriculum was to enable participants to act quickly on what they had learned and share their learning with family and community members to create further motivation for health service utilization, thus building trust in government health services.

The curriculum was pilot tested with a group of 15 learners during a weeklong facilitator training so that adaptations could be made to fit the needs of participants and facilitators.^19^^,^^21^ The curriculum was originally developed for 15–24-year-olds—the age range defined by the United Nations as “youth.” However, during pilot testing we learned that the likely age range of participants would skew older and that many would already be parenting. Although the Advancing Youth Project is open to younger participants, it tends to draw older participants who were denied schooling and now see that they need literacy and numeracy skills in order to better support themselves and their families, and to gain respect in the community. However, levels of SRH knowledge remain very low, regardless of age. We therefore added content to the curriculum that included positive parenting and parent-child communication about SRH.^31^

Skilled facilitators, called County Health Leads, were trained to deliver the curriculum. County Health Leads were PSI employees, trained as health educators, and chosen for their ability to communicate well with young adults in a fun, nonhierarchical, and yet still authoritative fashion. County Health Leads worked in pairs of one man and one woman. They were not teachers from the Advancing Youth Program—who were uncomfortable with and lacked knowledge to provide in-depth information about SRH—but rather knowledgeable outsiders brought into the program to engage topics that “insiders” usually cannot broach. County Health Leads visited a different USAID Advancing Youth Project site each week, in pairs, to implement the curriculum.

During the course of the *HealthyActions* week, the County Health Leads elected, with their permission, 2 learners to serve as Peer Health Educators. The learners were selected based on their demonstrated leadership and grasp of the curriculum, as well as eagerness to remain involved with the project after the 6-day intervention. Peer Health Educators volunteered to sustain the teachings of the program among their peer learners and other community members. Following *HealthyActions*, they received a formal 3-day training from the County Health Leads and PSI staff on delivery of the *HealthyActions* curriculum. However, because of low literacy levels, PSI and EDC first created 8 recordings that could be played through basic mobile phones. The recordings, produced in the style of popular Liberian radio shows and in Liberian English, feature a host who guides the Peer Health Educator through a facilitation of that recording’s lesson. For instance, the host says, “My friend [referring to the Peer Health Educator], please ask the group what they have heard about the oral contraceptive pill.” After a 2-minute pause, the host provides a brief explanation of the oral contraceptive pill. A sample recording can be found on PSI’s website.^31^ Peer Health Educators convened small gatherings within their communities to listen to and discuss the recorded lessons on the phones. Peer Health Educators also provided vouchers for family planning referrals to locally accessible clinics. Peer Health Educators used FrontlineSMS to send text messages to PSI and EDC on the number of participants, disaggregated by gender, in their meetings and the number of vouchers distributed.

*HealthyActions* was implemented twice between 2012 and 2014. The first round of implementation took place from August 2012 to July 2013 in Nimba and Montserrado counties as a pilot to gain feedback on the curriculum and approach. The second round of larger-scale implementation took place from November 2013 to May 2014 in Bong, Grand Bassa, Lofa, Montserrado, and Nimba counties. This second round included the Peer Health Educator component of the program. The evaluation for the program was undertaken during the second round of implementation.

### Evaluation Design

We used the Consolidated Standards of Reporting Trials (CONSORT) statement to structure reporting of this evaluation. A randomized design was used to evaluate the intervention during the second phase of *HealthyActions*. Eligible USAID Advancing Youth Project learning sites were identified as clusters to be randomized. Learning sites were randomly assigned to receive *HealthyActions* during the evaluation period or to serve as control sites. Learning sites were not eligible to participate if they were private education institutions and/or had participated in the first phase of *HealthyActions*. Out of 150 sites assessed for eligibility, 34 sites were assigned to the treatment group and 26 sites to the control group using a lottery process executed by PSI/Liberia research staff (Figure). Sample size was calculated using G*Power software version 3.1.6. The following parameters were used: difference between 2 independent means statistical test, *a priori* type of power analysis, one-tailed, .005 probability of Type I error, 95% power, 1/1 allocation ratio. Resulting sample size was 1,144, rounded to 1,200.

**FIGURE.**
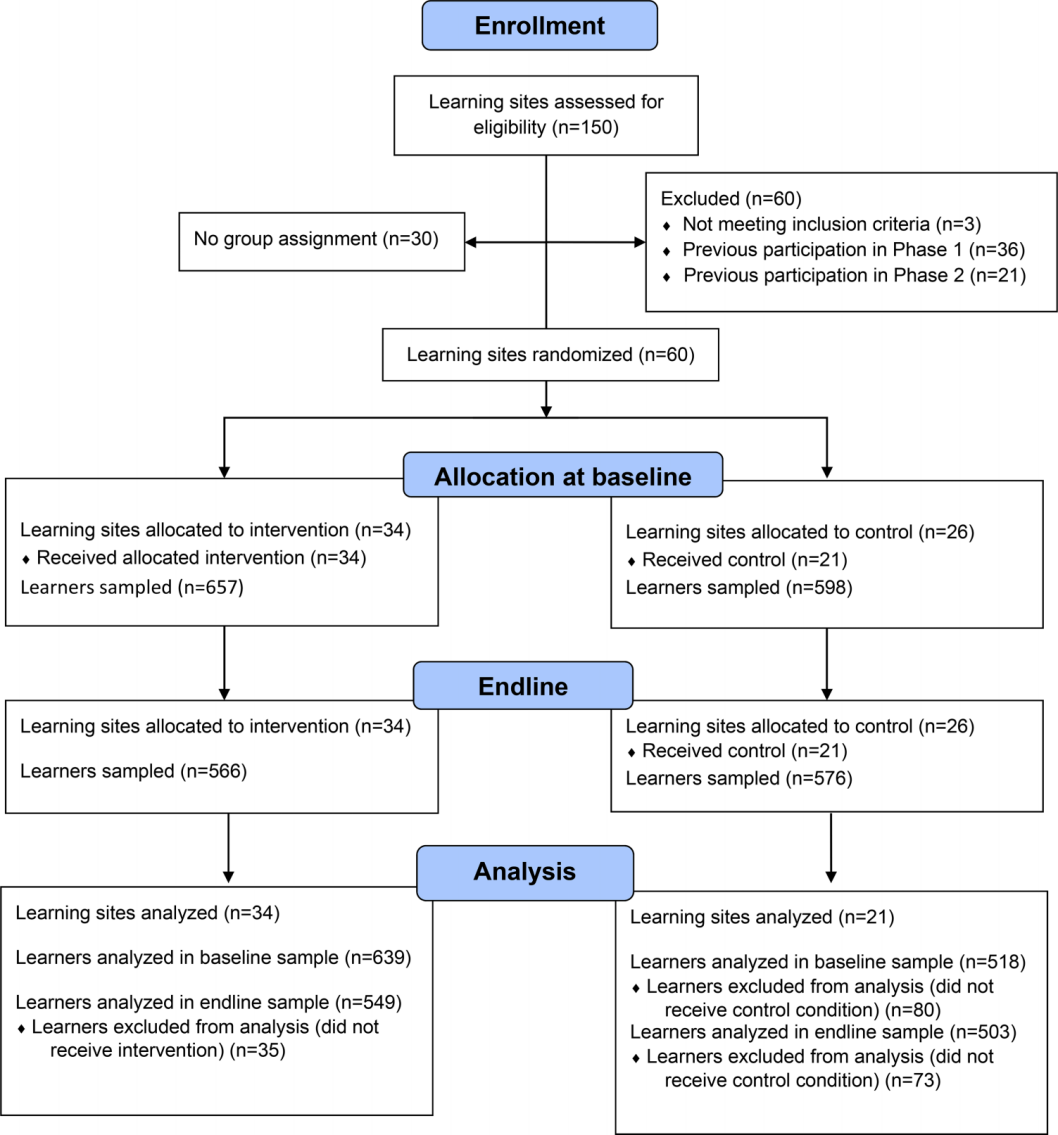
Flow Diagram of Enrollment, Allocation, and Analysis Process

The total number of eligible sites was stratified by county, and sites were sampled probability proportionate to the number of eligible sites per county. Site names were written on paper and stacked in separate piles. One county at a time, site names were placed in a box. Names of sites were selected from the box, with the first draw for treatment sites, the second for control sites, etc. One member of the research team selected treatment sites; another selected control sites; and a third held the box. Blinding of sites and participants to intervention status was not possible. The University of Liberia Institutional Review Board provided ethical approval for this study.

### Data Collection

Baseline data collection occurred in January–March 2014 and was conducted on a rolling basis across all 60 selected sites. Teams of data collectors and quality control supervisors were assigned by county, due to transportation challenges. At intervention sites, baseline data were collected on day 2 of the *HealthyActions* week, while at control sites baseline data were collected during standard USAID Advancing Youth Project sessions. Eligible study participants—current USAID Advancing Youth Project learners between the ages of 15–35—were randomly sampled from the group of learners available on the day that the data collection team visited a selected learning site. After informing all learners about the purpose and objectives of the evaluation, a minimum of 20 eligible learners per site were selected by asking learners to pull blocks out of a bucket. The number of blocks in the bucket corresponded to the number of learners at the site that day, with blocks coded for selection to participate in the evaluation. Learners could not see into the bucket when they selected a block. Identities of selected participants were cross-checked against a site roster after sampling.

After ensuring informed consent, trained female data collectors administered a 20–25 minute questionnaire on sociodemographic characteristics, parent-child and partner communication, gender norms, sexual history, contraception knowledge and use, HIV knowledge and testing experience, and, for female respondents, pregnancy history. The questionnaire was developed using Liberian English terms when possible, and data collectors were trained to administer the questionnaire consistently. Respondents above the age of 18 were administered a verbal consent script, and enumerators recorded their consent to participate via tablet. A paper consent form was administered to eligible respondents under the age of 18, with the minor’s informed assent, and consent provided by the learning site administrator in lieu of a parent. Data were collected electronically using SurveyToGo on Android tablets. Questionnaire administration occurred away from other learners, typically in an empty classroom.

Endline data collection occurred after all intervention sites completed *HealthyActions* and during standard USAID Advancing Youth Project sessions at both intervention and control sites in June–July 2014. Because of expected difficulties in contacting learners sampled at baseline and confirming their identities, the survey was administered to a newly selected sample of USAID Advancing Youth Project learners using the same sampling techniques as at baseline.

For this analysis, we removed cases from 5 learning sites (4 sites assigned to the control group received the intervention; 1 site was not included in the initial sampling frame). The final analytical sample included 55 sites.

### Measures

Outcome measures used for this analysis were as follows:

Among women:

1. Contraceptive use (modern or traditional methods)^32^

Among women and men:

2. Condom use with regular partners and with casual partners (never; intermittently, including sometimes or most of the time; or always)
3. HIV knowledge
4. Ever been tested for HIV and know the result
5. Intention to seek HIV counseling and testing in the next year

Treatment was defined as assignment to a learning site where *HealthyActions* was implemented. Other measures assessed included sociodemographic factors of age, gender, daily income (no income; 1–100 Liberian dollars [LRD]; 100–500 LRD; 600–1,000 LRD; or >1,000 LRD, where 85 LRD = US$1), alternative basic education learning level (1, 2, or 3), number of living children, and marital status (single/never married, married/living together, divorced, or widowed). We also assessed women’s reproductive history including number of pregnancies and live births, currently pregnant or breastfeeding, and reproductive intentions.

### Statistical Analysis

Descriptive statistics were calculated at baseline and endline. Balance between treatment and control was assessed at baseline using chi-square tests and *t* tests to assess quality of randomization. For all outcomes, a difference-in-difference logistic regression model with fixed effects was used to assess the effect of *HealthyActions* participation on observed changes in the outcomes of interest. The general model was specified as:


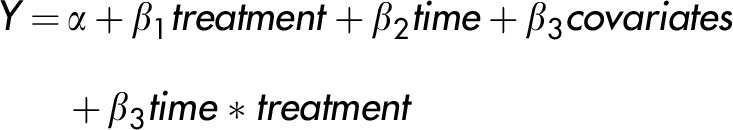


This model adjusted for both observed and unobserved differences between treatment and control at baseline to minimize possibility of confounding. The interaction term between treatment (coded 0 = control site and 1 = treatment site) and time (coded 0 = baseline and 1 = endline) served as the intervention effect. Other key demographic variables (learning level [1, 2, or 3], income, marital status, age, number of children) were included in the model to account for differences between the samples at endline. Interactions with age and gender were tested for all outcomes. Standard errors were clustered at the learning-site level to account for clustering due to randomization. All analyses were conducted using Stata 13.

## RESULTS

At baseline, a total of 1,255 learners were recruited to participate in the evaluation across 34 intervention sites and 26 control sites (Figure). At endline, 1,142 learners were recruited across the same number of learning sites. For this analysis, we assessed outcomes for 1,157 learners at baseline and 1,052 learners at endline in 55 learning sites. *HealthyActions* treatment and control groups shared similar demographic profiles at baseline and endline, indicating that randomization produced a balanced sample (Table 1). At baseline and endline, the sample consisted primarily of women with an average age of 28 years. The majority of respondents had a very low level of education (corresponding to Alternative Basic Education level 1), were married or living with a partner, and had children. At baseline, there were no statistically significant differences between the treatment and control groups on any demographic variables. At endline, the control group had slightly more women than the treatment group, and the control group was slightly more educated and had a slightly higher average income.

**TABLE 1:** Demographic Profile of Sampled Learners at Baseline (January–March 2014) and Endline (June–July 2014), Selected Counties of Liberia

	Baseline (N = 1,157)			Endline (N = 1,052)		
	Control (n = 518)	Treatment (n = 639)	*P* Value	Control (n = 503)	Treatment (n = 549)	*P* Value
Female, No. (%)	368 (71.04)	466 (72.93)	.48	327 (65.01)	400 (72.86)	.01
Age, years, mean (SD)	28.41 (5.93)	28.09 (5.81)	.35	28.03 (5.86)	28.86 (6.24)	.41
No. of children, mean (SD)	2.91 (2.08)	2.83 (2.16)	.55	2.73 (2.17)	2.79 (1.93)	.65
No. of children, No. (%)	65 (12.55)	77 (12.05)	.80	75 (14.91)	86 (15.66)	.73
Marital status, No. (%)			.70			.08
Single/never married	187 (36.10)	241 (37.72)		167 (33.2)	180 (32.79)	
Married/living together	313 (60.42)	379 (59.31)		320 (63.62)	340 (61.93)	
Divorced	14 (2.70)	12 (1.88)		6 (1.19)	20 (3.64)	
Widowed	4 (0.77)	7 (1.10)		10 (1.99)	9 (1.64)	
Educational level, mean (SD)	1.72 (0.82)	1.65 (0.73)	.12	1.78 (0.81)	1.60 (0.75)	<.01
ABE level 1, No. (%)	268 (51.74)	324 (50.70)		232 (46.12)	306 (55.74)	
ABE level 2, No. (%)	127 (24.52)	216 (33.80)		148 (29.42)	156 (28.42)	
ABE level 3, No. (%)	123 (23.75)	99 (15.49)		123 (24.45)	87 (15.85)	
Income, No. (%)			.10			.08
0	74 (14.29)	127 (19.87)		98 (19.48)	137 (24.95)	
LRD 1–100	80 (15.44)	83 (12.99)		52 (10.34)	70 (12.75)	
LRD 100–500	234 (45.17)	257 (40.22)		210 (41.75)	237 (43.17)	
LRD 600–1,000	68 (13.13)	77 (12.05)		79 (15.71)	57 (10.38)	
More than LRD 1,000	58 (11.20)	94 (14.71)		61 (12.13)	47 (8.56)	
Don’t know/no answer	4 (0.77)	1 (0.16)		3 (0.60)	1 (0.18)	

Abbreviations: ABE, alternative basic education; LRD, Liberian dollars; SD, standard deviation.

We saw modest improvements in condom use with a regular sexual partner after taking part in *HealthyActions* (Table 2). No statistically significant differences by age (15–24 vs. 25–35) or gender were found. After adjusting for potential confounders, individuals in the treatment sites were 12% less likely to report never using a condom with a regular partner over the last month when compared with the control group (*P* = .02). Positive changes in condom use were also noted among learners reporting that they use condoms always or intermittently, although these findings were not statistically significant. Frequency of condom use was slightly higher among learners reporting condom use with a casual partner, but at endline only 38% of those in the control group reported always using a condom with their casual partners compared with 44% of those in the treatment group. None of these changes was statistically significant.

We saw modest improvements in condom use with a regular sex partner after taking part in *HealthyActions*.

**TABLE 2:** Self-Reported Condom Use With Regular and Casual Partners Among Sampled Learners at Baseline (January–March 2014) and Endline (June–July 2014), Selected Counties of Liberia

	Baseline			Endline			Average Marginal Effects (95% CI)	*P* Value
	Control	Treatment	*P* Value	Control	Treatment	*P* Value		
With regular partner			.62			.01		
Sample size	356	427		312	304			
Never	229 (64.33)	288 (67.45)		199 (63.78)	159 (52.30)		-0.12 (-0.22, -0.02)	.02
Intermittently	102 (28.65)	114 (26.70)		94 (30.13)	113 (37.17)		0.08 (-0.02, 0.17)	.13
Always	25 (7.02)	25 (5.85)		19 (6.09)	32 (10.53)		0.04 (-0.01, 0.09)	.10
With casual partner			.24			.85		
Sample size	89	121		80	84			
Never	34 (38.20)	56 (46.28)		25 (31.25)	24 (28.57)		-0.05 (-0.18, 0.07)	.41
Intermittently	33 (37.08)	31 (25.62)		24 (30.00)	22 (26.19)		-0.4 (-0.16, 0.08)	.48
Always	20 (22.47)	33 (27.27)		30 (37.50)	37 (44.05)		0.10 (-0.07, 0.28)	.25
Don't know	2 (2.25)	1 (0.83)		1 (1.25)	1 (1.19)		–	–

Abbreviation: CI, confidence interval.

Data reported as No. (%) except when otherwise noted.

Among women who were not pregnant or breastfeeding at the time of the survey, substantial increases were seen in use of contraception for those in the treatment group (Table 3). Young women participating in *HealthyActions* were 13% more likely to report using a modern method of contraception than women in the control group (*P*<.01). Increases in modern contraceptive use were greatest among unmarried women (33.7 percentage points; *P* = .002), and among adolescents ages 15–19 we saw a 13 percentage point increase (*P* = .005). Specifically, participation in *HealthyActions* was associated with an 8 percentage point increase in the probability of using oral contraceptives (*P* = .06) and a 9 percentage point increase in the probability of using implants (*P* = .007), and there were no differential effects by age. In all groups before and after the intervention, injectables and pills remained the first and second most popular methods, respectively. Among the poorest women, at endline those in the treatment group were 31% (*P* = .009) more likely to report using an implant than those in the control group.

Young women participating in *HealthyActions* were 13% more likely to report using a modern contraceptive method than women in the control group.

**TABLE 3:** Current Contraceptive Use Among Non-Pregnant, Non-Breastfeeding Women at Baseline (January–March 2014) and Endline (June–July 2014), Selected Counties of Liberia

	Baseline (N = 546)			Endline (N = 470)			Average Marginal Effects (95% CI)^a^	*P* Value
	Control (n = 232)	Treatment (n = 314)	*P* Value	Control (n = 215)	Treatment (n = 255)	*P* Value		
Short-acting methods								
Injectable	56 (24.14)	76 (24.20)	.99	65 (30.23)	62 (24.31)	.15	-0.05 (-0.13, 0.04)	0.27
Male condom	19 (8.19)	29 (9.24)	.67	18 (8.37)	28 (10.98)	.34	0.03 (-0.0, 0.93)	.37
Pill	53 (22.84)	53 (16.88)	.08	33 (15.35)	60 (23.53)	.03	0.08 (0.00, 0.17)	.06
Female condom	1 (0.43)	0 (0.00)	.24	0 (0.00)	1 (0.39)	.21	–	–
Long-acting methods								
Implant	9 (3.88)	17 (5.41)	.41	16 (7.44)	39 (15.29)	.01	0.09 (0.03, 0.16)	<.01
Female sterilization	1 (0.43)	1 (0.32)	.83	0 (0.00)	0 (0.00)	>.99	–	–
Male sterilization	0 (0.00)	0 (0.00)	>.99	0 (0.00)	0 (0.00)	>.99	–	–
IUD	0 (0.00)	0 (0.00)	>.99	0 (0.00)	0 (0.00)	>.99	–	–
Any long-acting or permanent method	10 (4.31)	18 (5.73)	.63	16 (7.44)	39 (15.29)	.01	0.10 (0.05, 0.16)	<.01
Any modern method	123 (53.02)	159 (50.64)	.58	119 (55.35)	170 (66.67)	.01	0.13 (0.04, 0.22)	<.01
Traditional methods								
Rhythm	4 (1.72)	4 (1.27)	.67	8 (3.72)	7 (2.75)	.55	–	–
Other traditional	4 (1.72)	0 (0.00)	.02	1 (0.47)	3 (1.18)	.40	–	–

Abbreviations: CI, confidence interval; IUD, intrauterine device.

Data reported as No. (%) except when otherwise noted.

a Estimations for some methods couldnot be calculated because of the small number of users in the sample.

Substantial improvements were seen in HIV counseling and testing after participating in *HealthyActions* (Table 4). At endline, 42% of individuals in the control group reported having been tested for HIV compared with 88% in the treatment group. After adjusting for baseline probability and other potential confounders, participation in *HealthyActions* resulted in a 45% increase in the probability that an individual had ever been tested for HIV and knew the result (*P*<.001). There was no differential effect of the intervention for HIV testing among men versus women, nor by age. Intention to test was high in both groups at baseline; however, learners in treatment sites had a 6% higher probability than those in the control sites to report planning to get an HIV test in the upcoming year (*P*<.001).

Substantial improvements were seen in HIV counseling and testing after participating in *HealthyActions*.

**TABLE 4:** HIV Knowledge, Attitudes, and Testing Behaviors Among Sampled Learners at Baseline (January–March 2014) and Endline (June–July 2014), Selected Counties of Liberia

	Baseline (N = 1,157)			Endline (N = 1,052)			Average Marginal Effects (95% CI)
	Control (n = 518)	Treatment (n = 639)	*P* Value	Control (n = 503)	Treatment (n = 549)	*P* Value	
Have you heard of HIV or AIDS?	510 (98.46)	629 (98.44)	.99	498 (99.01)	547 (99.64)	.38	0.00 (-0.01, 0.02)
Do you know where to access HIV testing services?	366 (70.66)	423 (66.20)	.07	357 (70.97)	494 (89.98)	<.001	0.19 (0.13, 0.26)**
Have you ever been tested for HIV and received the result?	237 (45.75)	241 (37.72)	.001	211 (41.95)	482 (87.80)	<.001	0.45 (0.38, 0.53)**
Do you intend to get an HIV test within the next year?	474 (91.51)	560 (87.64)	.01	457 (90.85)	533 (97.09)	<.001	0.06 (0.03, 0.09)**

Abbreviation: CI, confidence interval.

Data reported as No. (%) except when otherwise noted.

* *P*<.05;

** *P*<.001.

Benefits of participating in *HealthyActions* were concentrated among youth under the age of 19, particularly related to knowledge of HIV and where to get tested. On average, youth under the age of 19 in *HealthyActions* sites were 32% more likely to know where to get an HIV test than their counterparts in the control sites (*P*<.001). A 3-way interaction term between assessment period, intervention exposure, and age was also found to be highly significant (*P* = .02), indicating that the intervention had a positive, differential effect on adolescents 15–19 years old compared with the general population of learners in the study. Girls under the age of 19 also showed increased knowledge of where to obtain an HIV test as a result of *HealthyActions*: adolescent girls in *HealthyActions* sites were 40% (*P*<.001) more likely to report knowing where to obtain an HIV test than their counterparts in control sites; in comparison, adolescent boys were 26% (*P* = .02) more likely to know where to obtain a test than their counterparts in control sites. A 4-way interaction term was also tested between assessment period, intervention exposure, age, and gender, which was also found to be statistically significant (*P* = .01), indicating that *HealthyAction*s had a stronger effect among girls than boys.

Benefits of participating in *HealthyActions* were concentrated among youth under the age of 19.

## DISCUSSION

We sought to assess the effectiveness of an intensive sexual and reproductive health intervention embedded in an alternative basic education program for Liberian out-of-school young adults. Combining intensive group learning with provision of on-site health services increased use of contraception and HTC in this setting. The *HealthyActions* group setting, educational components, and on-site access to services were designed, implemented and tested as a package; thus, we cannot tease out whether some components had greater impact than others nor whether some components could have been removed. We hypothesized that the components interacted with each other to achieve impact, but this could be formally tested in future research.

Combining intensive group learning with provision of on-site health services increased use of contraception and HIV testing and counseling services among Liberian youth.

Increases in use of contraceptive implants observed among the *HealthyActions* sites are likely related to the program’s emphasis on providing information and counseling to young women about long-acting reversible contraceptive methods as well as improving access to the products themselves. Implants are generally used less frequently than other methods in Liberia: 11% of currently married women in Liberia use injectable contraception and 2% use implants, while 22% of sexually active unmarried women use injectables and 4% use implants.^4^ We suspect that contraceptive implants were less familiar to *HealthyActions* learners. Many young women likely did not have sufficient information about implants from other sources, so supportive information about the method, provided in the context of informed choice, and with ready access to services, was an attractive offer. Further, contraceptive stock-outs are often widespread in Liberia, limiting access to the full range of methods. Easy access to a long-acting method likely helped to motivate women to choose implants. We suspect that assurances that the commodities would be available increased women’s confidence in and willingness to use the services provided on clinic celebration day. Counseling on removal of implants was also provided.

Despite the substantial increase in use of implants, *HealthyActions* did not appear to have an impact on use of long-acting or permanent methods overall. Increase in the use of implants corresponded with a decline in use of injectables among *HealthyActions* participants, although the decline was not statistically significant. It was not possible in this study to measure method switching, but it is possible that the availability of implants along with comprehensive information about all contraceptive methods contributed to a small substitution effect by which young women using injectables switched to implants. As implants provide a longer-term solution for women who want to avoid a pregnancy than do injectables, this finding is still consistent with program goals and the value of informed choice.

Increased use of implants corresponded with a decline in use of injectables among HealthyActions participants, suggesting a possible substation effect.

Continuation rates may ultimately improve among these women given that implants do not require resupply, which would also be a positive result of the study, although it is not possible to assess continuation with the data available. Longer-term follow-up would need to be conducted to assess whether continuation rates improved after *HealthyActions*, which was beyond the scope of this study.

The large proportion of *HealthyActions* learners who were tested for HIV is indicative of the existing barriers to accessing HTC in Liberia. The national average for HIV testing is 23% and 45% for men and women, respectively,^4^ whereas 88% of respondents in *HealthyActions* learning sites had been tested for HIV at endline and knew their results. The *HealthyActions* curriculum delivered factual information and sought to discredit myths and misconceptions about HIV in addition to supporting confidentiality of services. Due to the interconnectedness of many Liberian communities, protocols around confidentiality are not always respected. Many young Liberians don’t access health services, particularly those for HIV, for fear of being gossiped about by staff and older patients.^33^ *HealthyActions* may have also succeeded in forging a connection between learners and their local clinics or hospitals. Despite Liberia’s low HIV prevalence, *HealthyActions*’ large effect on use of HIV testing and counseling services holds promise for replication in other settings with higher HIV prevalence.

Condom use was featured throughout the *HealthyActions* curriculum, including activities that highlighted (1) the dual protective benefits of condoms (to prevent HIV and other sexually transmitted infections [STIs] as well as unintended pregnancy); (2) how to negotiate condom use particularly by young women; and (3) condom demonstrations and practice with models. There are a few possible explanations to account for the changes in condom use with regular partners that we observed. Trends were suggestive of improvements in intermittent or consistent use of condoms with regular partners. The decline in reporting never using condoms with a regular partner was statistically significant, but increases in use were not. Respondents may have struggled with distinctions between regular and casual partners given that regular partners are often loosely defined in a Liberian context, and regular may not equate with monogamous. According to the 2013 Liberian Demographic and Health Survey, 7% of women reported 3.4 sexual partners in the past year, and 18% of men reported 8 partners.^4^ *HealthyActions* did not touch on potential risks of concurrent partnerships, but the program encouraged consistent condom use and supported development of skills in partner communication and condom negotiation. An intervention of longer duration may be needed to address condom use with Liberian young adults. Further, additional research is needed to gain insight into sexual partner types and processes of partner formation in Liberia, including the influence of economic factors. Available evidence suggests that these processes are complex.^12^

We implemented *HealthyActions* within a specifically targeted environment of learning sites providing alternative basic education. The selection of this setting was deliberate in order to leverage a group setting that facilitated the formation of trusted peer networks. However, descriptive statistics of sampled learners were generally comparable to the rural Liberian population.^4^ *HealthyActions’* approach may have broader applicability to other settings where young adults, particularly those outside the formal school system, gather in Liberia or in similar settings, such as women’s associations, farmer groups, or trade associations. However, some specific subgroups of out-of-school youth, such as male ex-combatants, may need different intervention strategies.

As implemented in this setting, *HealthyActions* was designed as a short, intensive intervention to test the approach. However, the implementation model could be readily adapted to increase its scalability. *HealthyActions* could be delivered over the course of several weeks—with one activity per week for instance—rather than in 5 consecutive days. The curriculum could also be delivered as one intensive burst, with follow-up “booster” sessions every few months to sustain the messages. Additional studies could be used to measure the effectiveness of the short-term, intensive “burst” of education versus a longer-term, lower dosage program. Linkage to service delivery could also be adapted. In this setting, linking motivations and skills built into the curriculum to use of readily accessible services was important to enhance participants’ and community members’ trust in the health system. In other settings, delivery of the curriculum could be timed to coincide with regularly scheduled mobile health units, or the curriculum could provide vouchers or referrals to static health clinics, in either the public or private sector. Again in this setting, clinic celebration days included provision of contraception and HTC services. Antenatal and/or postnatal care could potentially be added. The components of the program promote ready adaptation and investigation to determine what a “lighter touch” yet still effective implementation model might be.

The HealthyActions model could be readily adapted to increase its scalability.

Our findings contribute to filling a gap in evidence for how to reach the large population of out-of-school youth and young adults in sub-Saharan Africa, who are otherwise engaged in a group learning setting, with comprehensive SRH information and services. A market-based intervention in Nigeria found similar increases in contraceptive use, but no change in condom use among sexually active youth.^34^ Community-based and structural interventions can contribute to attitudinal changes around sexual risk and partnership formation, and there is some evidence of reductions in risk of STIs.^35^ However, these interventions have tended to focus on STIs/HIV, without necessarily combining access to contraceptive services. Youth-friendly services can successfully provide HTC but are challenged to retain youth in follow-up care, a limitation of *HealthyActions* as well.^36^ However, we have not been able to locate programs that show comparable increases in levels of HIV testing uptake among African youth.

### Limitations

This study had several limitations. Because *HealthyActions* was designed as a package that combined a “burst” of learning with rapid provision of services, we cannot assess the relative effects of the components. However, the learning component was designed to motivate use of services, and thus attempting to tease out effects attributable to each component may not have been appropriate. Further, we were not able to assess any potentially beneficial “spill-over” of *HealthyActions* in uptake of services by community members, since only Advancing Youth learners were sampled. We initially intended to recruit and follow a sample of learners in intervention and control sites over time to control for individual preferences. However, we determined that tracking individuals over time would not be feasible due to challenges in ensuring that unique identifiers would remain unique. We therefore recruited cross-sectional samples at baseline and endline, using the same sampling procedure to ensure comparability. It is possible that our design was under-powered, since initial sampling plans did not fully account for clustering of learning sites. However, substantial effect sizes and narrow confidence intervals, even accounting for clustering of standard errors during analysis, suggest that we did have sufficient statistical power.

Due to the rolling nature of data collection, intervention sites received differing recall periods following implementation of *HealthyActions*, which may have attenuated estimates. Recall of some outcomes, such as condom use, may have been subject to social desirability bias.

The timing of endline data collection coincided with harvest season, rainy season, and the timing of tribal bush schools. As data collection took place during the early evening hours, the onset of the harvest season meant that some learners were needed on the farms until after dark. This was problematic because some learners would not be able to attend class until much later than usual and missed the sampling process. In an effort to address this issue, a small number of sites were resampled after those working on the farms arrived. In many parts of rural Liberia, participation in tribal bush schools marks the transition from childhood to adulthood. Often, the young people participating in the bush schools will be away for weeks at a time; this happened to coincide with endline data collection at one site, and the site was therefore resampled.

Training health service providers in youth-friendly services was outside the scope of this project, and therefore we could not guarantee that the health services we brought on the community celebration day would be aligned with standards for youth-friendly health services.^37^ PSI had since undertaken a training program for health care providers in the private sector in Monrovia to offer youth-friendly health services, particularly with adolescents and younger, unmarried youth in mind, and this program ran concurrently to the second round of *HealthyActions*. Funds were originally allocated for scale-up of youth-friendly health services; however, they were redirected to Ebola efforts. The County Health Leads did meet with the government health care providers before the program to prepare them for the community celebration day and confirm their participation and mitigate against the risk of unfriendliness during the intervention.

## CONCLUSION

Despite a range of obstacles to progress for Liberia’s young people, the desire for opportunities for learning and growth is ever-present. *HealthyActions* brought together key components of established social ties, intensive learning, and convenient SRH services, presenting a new opportunity for many people in need. Based on our findings, this comprehensive package targeted to young adults appears to have produced substantial results; expanding access resulted in increased uptake of both modern contraceptives, particularly implants, and HIV counseling and testing.

The concentration of positive family planning results among youth under 19 and the success in increasing uptake of contraceptive implants suggests that the *HealthyActions* model is successfully affecting the portion of the target audience that is most difficult to reach and that stands to gain the most from using contraception.

*HealthyActions* targets youth who are unlikely to be reached by traditional health outreach efforts, and holds promise to enact substantial behavior change over a short period of time. In a context where SRH knowledge and youth-friendly services are nearly nonexistent, getting the conversation started and making services available were the highest priorities of the program. Follow-on funding was sought and is considered to be crucial to institutionalizing the program and sustaining longer-term behavior change. In Liberia, this became challenging due to staff turnover and the need to respond to the Ebola epidemic. However, the program’s focus on sexual and reproductive health integration within an established learning environment, particularly among low-literacy populations, presents an adaptable solution for health programming across the country and the region.

## Notes

**Peer Reviewed**
